# Piezoelectric sterilization techniques: from innovations to applications

**DOI:** 10.3389/fchem.2025.1709575

**Published:** 2025-11-20

**Authors:** Bidisha Ghosh, Zia Ullah, Tehseen Sehar, Subhasis Sarkar

**Affiliations:** 1 Department of Biotechnology, School of Life Sciences, Swami Vivekananda University, Kolkata, India; 2 School of Science, Harbin Institute of Technology, Shenzhen, Guangdong, China; 3 Department of Microbiology, School of Life Sciences, Swami Vivekananda University, Kolkata, India

**Keywords:** antimicrobial activity, biofilm, piezoelectric materials, piezodynamic therapy, reactive oxygen species (ROS), piezoelectric sterilization

## Abstract

Piezoelectric materials have emerged as promising non-thermal, chemical-free sterilization agents, offering clear advantages over traditional methods such as heat, UV, or disinfectants. Their antimicrobial activity arises from direct microbial membrane disruption and reactive oxygen species (ROS) generation under mechanical stimuli like ultrasound or vibration via piezodynamic reactions. These approaches preserve material integrity, making them ideal for implants, wound dressings, and biofilm prevention. Recent advances focus on enhancing piezoelectricity via defect engineering, dopants, and band structure optimization to improve ROS production. This review highlights progress in piezoelectric materials as smart, sustainable antimicrobial platforms with broad biomedical and environmental applications.

## 1 Introduction

Sterilization of material surfaces has remained a cornerstone of public health and industrial safety since ancient times, serving as a critical strategy to combat contamination and infection. A major driver of microbial persistence and dissemination is the formation of biofilms that not only enhance survival but also contribute to recurrent contamination and antibiotic resistance (Flemming et al., 2023). Cross-contamination from food, water, surfaces, and medical equipment remains a leading cause of microbial colonization and subsequent infections, which pose significant risks in both community and hospital environments. The conventional sterilization techniques, including moist heat autoclaving, dry heat, radiation, filtration, and chemical disinfectants, have long been indispensable in infection control. Among them, autoclaving has been the gold standard owing to its reliability and non-toxic nature, using saturated steam at 121 °C for 15–30 min to inactivate microorganisms, including resilient spores, through protein denaturation and enzyme inactivation. Other modalities, such as ionizing and non-ionizing radiation (Bisht et al., 2021), ultrafiltration (Barber et al., 2023; Holland et al., 2020), and chemical agents like ethylene oxide (Barwell and Freeman, 1959), acids, alkalis (Diez-Gonzalez et al., 2000), and chlorine (Natan et al., 2015), have been widely applied. However, these methods are often resource-intensive, requiring substantial energy, costly infrastructure, and prolonged processing times. Furthermore, some methods generate toxic byproducts with carcinogenic potential. For instance, chlorination may yield harmful disinfection byproducts, and UV irradiation requires extended exposure for reliable sterilization. These challenges are compounded in low-resource settings, where reliance on fossil fuel-derived energy exacerbates environmental concerns (Chen et al., 2024).

Given these limitations, there is a pressing need for alternative, eco-friendly, and efficient sterilization strategies. In recent years, piezoelectric materials have emerged as a new class of smart antimicrobials. A particularly exciting development is piezodynamic therapy (PZDT), which leverages piezocatalysis, the conversion of mechanical energy into electrochemical activity, to generate localized reactive oxygen species (ROS) under mechanical stimulation such as vibration, ultrasound, or shear stress. Unlike conventional antibiotics or harsh disinfectants, piezoelectric approaches are non-thermal, chemical-free, and controllable, reducing the risk of resistance development and minimizing environmental impact. The antimicrobial action of piezoelectric materials primarily stems from two mechanisms: (i) direct membrane disruption leading to cytoplasmic leakage and microbial death, and (ii) ROS-mediated oxidative stress caused by species such as hydrogen peroxide (H_2_O_2_), superoxide anion (⋅O_2_^−^), and hydroxyl radicals (⋅OH). Additionally, piezoelectric materials can be incorporated into coatings and composites to create reusable, self-sterilizing surfaces capable of continuous protection against microbial colonization (Soe et al., 2025). These characteristics make them particularly suitable for sterilizing heat-sensitive biomedical implants, surgical instruments, wound dressings, and filtration membranes.

Recent research emphasizes strategies to enhance piezoelectric performance for antimicrobial use. Defect engineering (Wang et al., 2022c), band structure tuning, and dopant incorporation have been employed to increase charge separation efficiency and ROS generation, thereby amplifying sterilization outcomes. These modifications provide tunability, enabling researchers to balance ROS production with ROS scavenging, optimizing antimicrobial efficiency while avoiding unintended cytotoxic effects (Soe et al., 2025). The appeal of piezoelectric sterilization lies in its sustainability, controllability, and broad applicability (Wang et al., 2022b). Unlike chemical disinfectants, it leaves no harmful residues; unlike antibiotics, it does not drive genetic resistance (Blair et al., 2015; Furuya and Lowy, 2006), and unlike thermal sterilization, it preserves the structural and functional integrity of sensitive materials. Despite significant advances, the field remains relatively nascent, with much scope for systematic exploration. This review seeks to fill that gap by presenting a holistic overview of recent developments in piezoelectric sterilization. It highlights advances in molecular and cellular mechanisms of PZDT-induced sterilization and biomedical device integration, offering perspectives on the future of piezoelectric materials as sustainable and intelligent sterilization platforms (Table 1).

**TABLE 1:** Applications of piezoelectric materials for antibacterial sterilization.

Survival strategies of microorganisms against conventional antibiotics			
Antibiotics	Resistant Bacteria	Mode of action	Ref.
Fluoroquinolones, βlactams, chloramphenicol, sulphonilamide	Enterobacteriaceae, *Pseudomonas* spp, *Acinetobacter* spp	Efflux Pumps, Enzymatic Hydrolysis	Papkou et al. (2020)
MLS^a^	Micromonospora sp, *Staphylococcus* *E. coli*	Degradation and Modification by hydrolytic enzymes like phosphotransferases, esterases, and hydrolases	Ounissi and Courvalin. (1985)
Aminoglycosides	*Campylobacter*	Modification of antibiotics by enzymes -acetyl transferase, Phosphotransferase, nucleotidyltransferase	Blair et al. (2015)
Linezolid	*S. aureus*	23S rDNA, Acetyl transferase enzyme	Roy et al. (2023)
Trimethoprim	*E. coli*	Mutations in the DHFR^b^ gene	Toprak et al. (2012)

Effect of ultrasound frequency on the inactivation of bacteria					
Piezocatalyst	Ultrasonic Frequency	Time	Microorganism	Inactivation Efficiency	Ref.
BaTiO_3_	40 KHz	180 min	*E. coli*	2.72 log	Feng et al. (2018)
PVDF@Ag@LiNbO_3_	40 KHz	180 min	*E. coli* ^a^, S. aureus^b^	5 log^a^, 1.47 log^b^	Singh et al. (2021)
Al_2_O_3_	40 KHz	120 min	*E. coli* ^a^, B. subtilis^b^	3.10 log^a^, 2.90log^b^	Zhao et al. (2022)
SGDY	—	—	*E. coli*	5log	Zhang et al. (2022)

A comparison of different piezo-photocatalysts in the degradation of organic pollutants					
Catalyst	Substrate	% Of Degradation	Time of Reaction	Catalytic Conditions	Ref.
CuS/ZnO nanowires	MB	99%	20 min	Xe Lamp + US	Hong et al. (2016)
AI/BaTiO_3_	4NP	100%	40 min	Xe Lamp + Vibration	Guo et al. (2019)
BiOI/ZnO nanorod array	Bisphenol A	100%	30 min	Visible Light + US	Zhang et al. (2020)
Rh-SrTiO_3_	Bisphenol A	77%	60 min	Visible Light	Zhou et al. (2020)
FTO/BTO/Ag NPs	CIP + MB	CIP (72%), MB (98%)	180 min	UV + US	Masekela et al. (2022)
BiVO4	MB	81%	240 min	Visible Light + US	Kumar et al. (2019)
NaNbO_3_/WO_3_ nanowires	RhB	73.7%	120 min	Visible Light + US	Yan et al. (2023)

Comparison of different piezo-photocatalysts in the degradation of bacteria					
Piezo-photocatalyst	Microorganism	% of Cidal activity	Time of Reaction	Catalytic Condition	Ref.
Au MoS2 NSs	*E. coli*	99%	15 min	Near IR + US	Chou et al. (2019)
BaTiO_3_	*E. coli* JM109	100%	30 min	UV + Vibration	Kumar et al. (2019)
BaZr0.02Ti0.98O3	*E. coli*	95%	90 min	UV light	Sharma et al. (2020)
BiVO4	*E. coli* ^1^ & *S. aureus* ^2^	90.44%^1^, 97.24%^2^	120 min	Visible Light	Deka et al. (2022)
g-C_3_N_4_/PMA	*S. aureus*	94%	180 min	Visible Light + US	Ammar et al. (2024)
SnO2/SnS2	S. mutans (in teeth) and Biofilms	77.3%, 56.5%	—	Yellow light + US	Song et al. (2025)

N.B.^a^MLS (Macrolide, Lincosamide, Streptogramin Type B), DHFR^b^ (Dihydro Folate Reductase enzyme), SGDY: Sulphur-doped graphydiyne, MB (Methylene Blue), 4NP (4 Nitrophenol), CIP (Ciprofloxacin), RhB (Rhodamine B), US (Ultrasonication), NS: nanosheets, PMA: phosphomolybdic acid, UV: ultraviolet.

## 2 Molecular and cellular mechanisms of PZDT-induced sterilization

### 2.1 ROS-mediated oxidative stress

ROS play a pivotal role in the antimicrobial mechanisms of piezoelectric materials. Upon mechanical stimulation, such as ultrasound, vibration, or fluid shear, piezoelectric crystals, including BTO, BiFeO_3_ (BFO), and NaNbO_3_, generate internal electric fields due to charge separation. The separated electrons and holes migrate to the material surface, where they react with dissolved oxygen and water molecules to produce diverse ROS, primarily ⋅OH, ⋅O_2_^−^, and H_2_O_2_ (Zeng et al., 2024). These ROS exert strong oxidative stress on microbial cells by simultaneously targeting critical biomolecules. The lipid bilayer is one of the earliest and most vulnerable sites of attack. The ⋅OH radicals initiate lipid peroxidation, compromising membrane integrity, increasing permeability, and causing uncontrolled leakage of cytoplasmic contents (Roy et al., 2025; Shang et al., 2025). Proteins and enzymes are oxidized at sensitive amino acid residues, disrupting metabolic activity, inhibiting energy production, and impairing key biosynthetic pathways. Furthermore, nucleic acids suffer oxidative base modifications, single- and double-strand breaks, and crosslinking, overwhelming the microbial DNA repair systems. Collectively, these effects lead to irreversible damage to structural and functional cellular components (Liu et al., 2024).

The outcome of this oxidative assault is either necrotic lysis or apoptosis-like microbial death. Several studies confirm this phenomenon: BFO-chitosan composites under ultrasound generated abundant ⋅OH radicals, eradicating *S. aureus*, while Ti-doped BTO in fibrous scaffolds disrupted bacterial membranes and induced nucleic acid leakage (Wu et al., 2024). Importantly, ROS generation occurs without external light or chemical additives, making piezoelectric sterilization a sustainable, controllable, and highly effective strategy against multidrug-resistant pathogens.

### 2.2 Membrane depolarization and electrostatic damage

In addition to ROS-mediated oxidative stress, membrane depolarization and electrostatic disruption constitute a second major mechanism through which piezoelectric materials inactivate microorganisms. The piezoelectric materials can influence the membrane potential (V_m_) through several interconnected mechanisms. When a piezoelectric material such as BTO, BFO, and PVDF is mechanically stimulated by ultrasound, vibration, or shear forces, it directly generates localized electric fields or surface charges by virtue of the piezoelectric effect. If a cell membrane is in proximity or adherent to that surface, the altered extracellular potential may shift the resting V_m_, modulating the electrostatic environment of the membrane. These fields arise from polarization charges exceeding physiological transmembrane potential thresholds, often ranging from 200 to 1,000 mV, and also disturb the microbial ionic homeostasis (Feng et al., 2018). For example, the influx of Ca^2+^ through voltage-gated calcium channels or Piezo1 channels, thereby changing ionic fluxes and directly altering the membrane potential through depolarization or hyperpolarization (Chen et al., 2024; Tsikriteas et al., 2023; Zheng et al., 2024). At lower intensities, reversible pore formation may occur, allowing ion flux and leakage of small molecules. However, stronger fields or prolonged exposure result in irreversible electroporation. This leads to large-scale cytoplasmic leakage, electrolyte imbalance, and eventual cell lysis. For example, PVDF-hydroxyapatite nanowire films subjected to ultrasound generated voltages up to ∼2 V, producing nanoscale pores in bacterial membranes and causing extensive protein and nucleic acid leakage (Dolai et al., 2023).

Electrostatic interactions further contribute to antimicrobial action. Bacterial cell walls, typically negatively charged due to teichoic acids (Gram-positive) or lipopolysaccharides (Gram-negative), are attracted to positively polarized piezoelectric surfaces. This adhesion enhances charge transfer and facilitates local depolarization events, accelerating membrane disruption. Additionally, these localized charges interfere with mechanosensitive ion channels and transporters, leading to downregulation of Na^+^/glucose symporters and respiratory proteins, thereby impairing energy metabolism. The combined effects of electroporation, cytoplasmic leakage, and electrostatic interference compromise microbial viability even in biofilm-associated cells (Shang et al., 2025). Notably, tetragonal BTO-based Janus nanomotors activated by ultrasound penetrated biofilms and induced both localized electric field damage and nitric oxide release, achieving efficient eradication of drug-resistant bacteria. These mechanistic pathways highlight that the effect of piezoelectric materials on membrane potential is often indirect, mediated by local electric fields, mechanical deformation, ion-channel activation, and substrate-cell interface effects, rather than simply applying a fixed voltage across the membrane. Thus, membrane depolarization and electrostatic damage represent a powerful, complementary mechanism to ROS-mediated stress in piezoelectric sterilization (Zhao et al., 2024).

### 2.3 DNA/RNA damage and replication arrest

**FIGURE 1:**
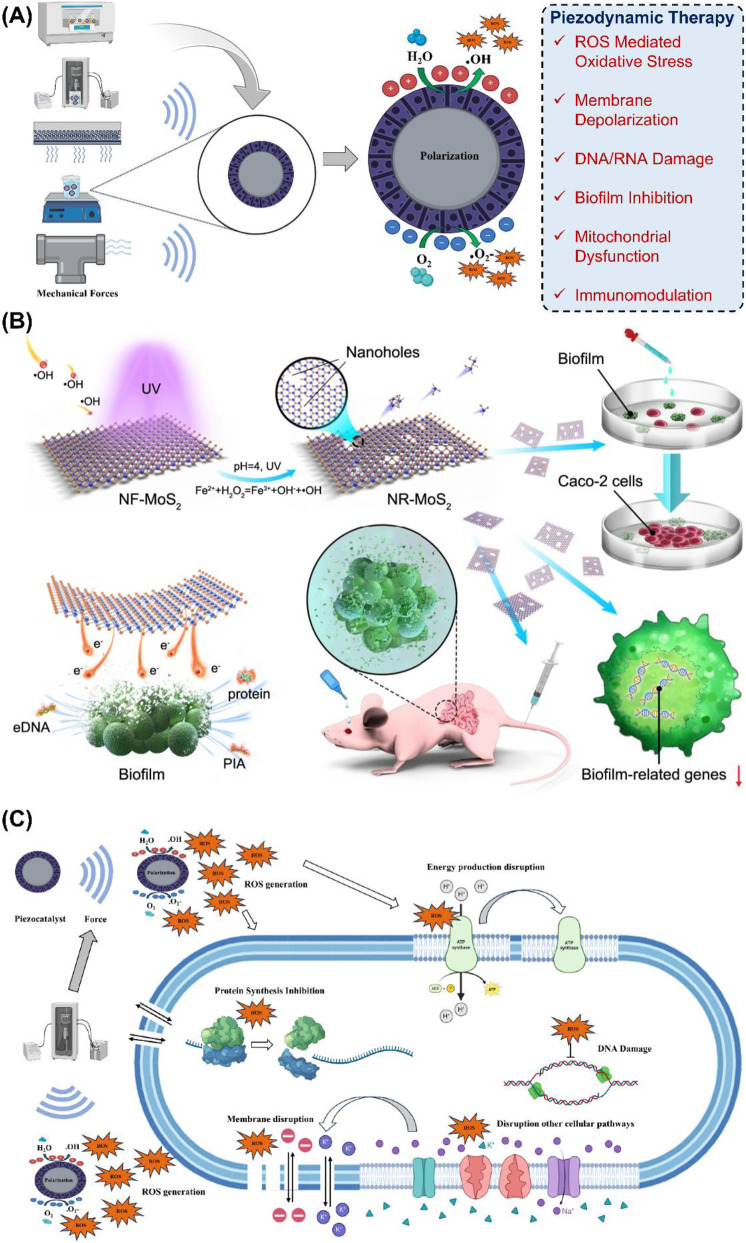
**(A)** Schematic representation showing how mechanical forces generate piezoelectric polarization in a piezoelectric material, subsequently triggering ROS-generating reactions on its surface. Adopted with permission from (Qurbani et al., 2025) under CC BY NC ND license. Copyrights 2025, Elsevier. **(B)** Downregulation of biofilm-forming genes by introduction of nanoholes in MoS_2_. The binding of *S. aureus* to Caco_2_ was reduced along with cell apoptosis. Adopted with permission from (Shi et al., 2021) under CC BY license. Copyrights 2021, Nature. **(C)** Piezocatalysis event showing membrane disruption due to ROS production and ultimate intracellular damage brought about by alteration and disruption of cellular and metabolic pathways, with concomitant DNA damage and protein inhibition. Adopted with permission from (Qurbani et al., 2025) under CC BY NC ND license. Copyrights 2025, Elsevier.

Beyond membrane disruption and oxidative stress, piezoelectric sterilization exerts profound effects on microbial genetic material, leading to DNA/RNA damage and replication arrest. When piezoelectric materials such as BTO, BFO, or MoS_2_-based heterojunctions are mechanically stimulated, they generate localized electric fields and abundant ROS (Figure 1). These factors synergistically target nucleic acids, producing irreversible genotoxic stress. ROS such as ⋅OH attack the sugar-phosphate backbone of DNA, causing single-strand breaks (SSBs), double-strand breaks (DSBs), and oxidative base lesions including eight-oxo-guanine, thymine glycols, and formamidopyrimidines (Berquist and Wilson III, 2012). Clusters of such lesions often overwhelm DNA repair systems, leading to chromosomal instability. Additionally, electric fields promote the formation of DNA-protein and interstrand cross-links, further obstructing transcription and replication. Unless repaired by base excision or homologous recombination pathways, these damages result in cell cycle arrest and eventual death. Equally significant is the inhibition of replication and transcription machinery. ROS oxidize thiol groups in essential enzymes such as DNA polymerases, RNA polymerases, and transcription factors, rendering them inactive. This halts DNA synthesis, prevents mRNA transcription, and blocks protein production (Zheng et al., 2025). Biofilm-forming bacteria exposed to piezoelectric nanocoatings exhibit extracellular DNA degradation and suppressed gene replication, weakening biofilm resilience and horizontal gene transfer.

Collectively, DNA/RNA damage and replication arrest disrupt microbial heredity and survival. By targeting genetic integrity and transcriptional processes, piezoelectric sterilization ensures irreversible inactivation, even in drug-resistant pathogens, positioning it as a powerful strategy beyond conventional antimicrobial approaches (Yi et al., 2025). Moreover, it has to be understood that resistance mechanisms of bacteria mainly arise through the application of chemical-mediated sterilizing agents, notably antibiotics, where modification of drug targets is possible. However, piezoelectric material-driven sterilization is typically a physical method of sterilization where ROS production is typically achieved through physical stimulus ranging from mechanical, electrical, and magnetic energies. This ROS production affects the potential of the cellular envelope of the bacteria, ultimately leading to cellular leakage rather than directly penetrating the cellular interior (Gazvoda et al., 2022).

### 2.4 Inhibition of biofilm formation and quorum sensing

Biofilms are structured microbial communities encased in an extracellular polymeric substance (EPS) matrix, which enhances adhesion and confers 10 to 1000-fold higher antibiotic tolerance compared to planktonic cells (Mah and O'Toole, 2001). They are implicated in 65% of nosocomial infections and facilitate horizontal gene transfer, promoting antibiotic resistance. The piezoelectric materials like BTO, BTO@Au, BiOI/Ti_3_C_2_, and MoS_2_ generate ROS through surface charge separation, mimicking mitochondrial electron transport processes. ROS degrade EPS components, including polysaccharides, proteins, lipids, and extracellular DNA, increasing matrix porosity, dispersing embedded bacteria, and enhancing antibiotic access (Figure 1). These effects have been demonstrated against MRSA, *E. coli*, *S. aureus*, and *C. albicans in vitro* and *in vivo* (Cao et al., 2025; Chen et al., 2025; Yu et al., 2023).

Piezoelectric-induced ROS also interfere with quorum-sensing (QS) systems, including AI-2 and LuxR-type pathways, by chemically degrading autoinducers and disrupting bacterial redox homeostasis (Amoils, 2005). This suppresses QS-regulated behaviors such as biofilm formation, swarming motility, and virulence factor expression. Mechano-biochemical effects from piezoelectric surfaces further suppress virulence gene expression and biofilm-associated resistance. For instance, Janus nanomotors (Zhao et al., 2024), PVDF-TrFE, BTO coatings, and BaCaTiZrO_3_ ceramics produce localized ROS, nitric oxide, and surface charges that compromise biofilm integrity, damage structural proteins, induce membrane depolarization, and downregulate biofilm-promoting genes (Shang et al., 2025). Collectively, piezoelectric materials offer a multifaceted, non-antibiotic approach to eradicate biofilms, attenuate bacterial virulence, and reduce chronic infection burdens.

### 2.5 Mitochondrial dysfunction in fungi and eukaryotic pathogens

The ROS, such as ⋅O_2_^-^ and ⋅OH, disrupt mitochondrial function by collapsing the mitochondrial membrane potential (ΔΨ_m_), opening permeability transition pores, and impairing ATP synthesis, ultimately causing pathogen death. This ROS-mediated mitochondrial dysfunction also facilitates cytochrome c leakage into the cytosol, initiating apoptosome-like complexes that activate caspase or metacaspase cascades, including caspase 9 and caspase 3, leading to DNA fragmentation and programmed cell death (Li et al., 2020).

In fungal cells, piezoelectric-induced ROS suppresses proliferation by disrupting respiratory processes and redox balance, damaging proteins, membrane lipids, and DNA, and inducing cell cycle arrest. Oxidative stress further triggers mitophagy and mitochondrial pore opening, activating caspase-like metacaspases that drive programmed cell death (Xu et al., 2023). Overall, piezoelectric materials exploit mechanotransduction to produce ROS, simultaneously inhibiting pathogen growth and activating intrinsic cell death pathways, representing a highly effective antifungal and antiparasitic strategy.

### 2.6 Immunomodulatory pathways in host-microbe context

The functional plasticity of macrophages, known as macrophage polarization, allows them to adopt pro-inflammatory M1 or anti-inflammatory/regenerative M2 phenotypes in response to environmental stimuli. Piezoelectric materials, which generate electrical charges under mechanical stress, significantly influence macrophage polarization. Materials like BTO, PVDF, ZnO, and their composites (e.g., GO-BTO) can modulate immune responses by surface charge generation, nanoscale morphology, and crystal orientation. Ultrasound-activated BTO nanoparticles enhance M1 polarization by increasing ROS, altering morphology, and upregulating TNF-α, IL-1β, IL-6, and iNOS, thereby boosting phagocytic and antibacterial activity via PI3K/Akt, MAPK, and FcγR pathways (Ganguly et al., 2024). In infection-repair contexts, these materials support M2 polarization, upregulating arginase-1, CD206, and anti-inflammatory cytokines (IL-4, IL-10), promoting tissue regeneration. Mechanistically, piezoelectric cues drive metabolic reprogramming, including fatty acid oxidation and amino acid biosynthesis, facilitating mitochondrial biogenesis for M2 development. Activation of mechanosensitive Piezo1, alone or with TLR4, also enhances Ca^2+^ influx, F-actin remodeling, and NFκB signaling, reinforcing M1 polarization during microbial challenges. Thus, piezoelectric stimulation enables dynamic immunomodulation, balancing M1 and M2 phenotypes to optimize host defense and tissue repair (Ijaz et al., 2024; Zhao et al., 2023).

## 3 Advanced applications and emerging concepts

### 3.1 Self-sterilizing biomedical devices

Piezoelectric materials are transforming biomedical implants and wound care by enabling self-sterilizing, regenerative surfaces through mechanically induced ROS and piezoelectric signals. In implants such as pacemakers, catheters, and orthopedic devices, piezoelectric coatings generate surface charges and ROS under ultrasonic or mechanical stimulation (Wang et al., 2025). These ROS disrupt bacterial membranes, degrade proteins and DNA, and interfere with extracellular polymeric substances, preventing biofilm formation and achieving over 90% antibacterial efficacy. In orthopedic applications, piezoelectric coatings also enhance osseointegration by producing endogenous electric fields that stimulate bone growth while simultaneously sterilizing the implant surface, combining infection control with regenerative promotion (Shang et al., 2025).

Dynamic piezoelectric dressings convert everyday mechanical energy, movement, respiration, or friction into continuous ROS and mild electric fields. Hydrogel-based dressings leverage this effect to suppress bacterial colonization, disrupt biofilms, and promote wound healing via collagen deposition, angiogenesis, and cell proliferation (Wei et al., 2025). Routine motion can also modulate fibrotic responses, guiding tissue toward regenerative repair. Collectively, piezoelectric implants and dynamic dressings offer smart, self-activating platforms that integrate antimicrobial defense with tissue regeneration, representing a new frontier in infection-resistant, multifunctional biomedical devices (Vijayakanth et al., 2023).

### 3.2 Wearable and on-skin electronics

Self-powered piezoelectric hydrogel patches convert biomechanical motion into localized electrical stimulation, mimicking endogenous electric fields to promote wound healing. These patches enhance re-epithelialization, angiogenesis, and collagen deposition, and modulate inflammatory signaling, shifting macrophages from M1 to M2 phenotypes (Wang et al., 2022a). In diabetic models, PVA/PVDF hydrogels and electrospun PLLA@Ga membranes improved fibroblast migration, growth factor secretion, and antibacterial clearance via ROS generation (Liu et al., 2025). 3D-printed ZnO-PVDF PiezoGels also accelerate closure and provide antimicrobial effects. These systems deliver continuous, self-powered electrotherapy for infection control and tissue regeneration (Vijayakanth et al., 2023). Moreover, the self-powered patches are used in the treatment of atopic dermatitis, which empowers a piezoelectric generator in a self-wearable patch that measures skin hydration. Liang et al., 2025 reported a self-powered patch, presenting a viable way to improve clinical results and treatment compliance by combining targeted medication delivery, self-powering capabilities, and real-time skin hydration monitoring. In a preclinical mouse model, the capacity of the patch to dramatically reduce epidermal thickness, prevent mast cell infiltration, and lower the expression of inflammatory mediators, particularly IL-4, demonstrated its therapeutic efficacy. The comfort and flexibility of the patch further highlighted its potential for everyday use, making it a good choice for the long-term treatment of persistent skin disorders (Liang et al., 2025). Recent reviews have pointed out the benevolence of piezoelectricity being used in cardiac patches. For example, Monteiro *et al.* showed that piezoelectric cardiac patches can be applied directly to living cardiac tissue without affecting its functionality and improve the molecular, cellular, and functional aspects of infarcted hearts. Piezoelectric patches improved electrical integrity at the functional level. The reduced LV mass and internal diameter in systole, as well as the reduced cross-sectional area of the cardiomyocytes, demonstrated that the piezoelectric patch enhanced cardiac tissue remodeling at the cellular and tissue levels. Far from the location of the injury, mice implanted with piezoelectric patches displayed downregulation of genes linked to ECM remodeling and overexpression of genes linked to mitochondrial production at the molecular level. These changes suggested the onset of tissue repair processes by the use of piezoelectric patches (Monteiro et al., 2025). In summary, these self-powered piezoelectric wearables represent a highly promising convergence of biomaterials, bioelectric stimulation, and wearable technology for enhancing tissue regeneration and managing chronic conditions.

### 3.3 Piezoelectric nanogenerators in hospital surfaces

Typically, the piezoelectric sterilization involves piezocatalysis by the mechanical stimulation of piezoelectric materials, leading to generate surface polarization. Consequently, this may generate ROS, which potentially kills microbes present on the surface. As goes with the conventional mode of sterilization, which is achieved either by exposing to physical stimulus (heat) or by spraying chemicals that readily may come into contact with humans and animals. Thereby, the direct contact of the chemicals at higher concentrations may lead to severe adversity to the skin of mammalian cells. Piezoelectric polymers offer touch-activated, chemical-free sterilization for high-contact surfaces like door handles and bedrails. Mechanical stimulation, mimicking human touch, induces surface charges and, in some cases, ROS, creating electrostatic and oxidative microenvironments that damage bacterial membranes and inhibit adhesion (Shang et al., 2025). PVDF nanoparticles effectively eliminated *E. coli* and reduced *S. aureus* under dynamic conditions, while nanotextured PLLA films achieved bactericidal effects without harming human cells (Fernandes et al., 2025). This mechanotransduction-based approach allows routine contact to trigger sterilization, reducing dependence on active cleaning and offering a practical strategy to limit fomite-mediated infection in healthcare and public environments.

It has to be noted that the ROS produced as a result of mechanical stimulation is selective towards the microorganisms and does not involve any potential harm to the host mammalian cells. Mention has to be made about reports cited by Zhao *et al.,* where the application of BTO composite (BTO@PDA-La) demonstrated excellent eradication of not only biofilm, but also achieved piezoelectric material accelerated wound healing through proper regulation of inflammatory factors (Zhao et al., 2024).

### 3.4 Air, water, and food sterilization systems

Recent piezoelectric membranes offer self-powered, mechanically activated solutions for pathogen removal in air, water, and soil. PLLA nanofibrous membranes maintain surface charges for electrostatic adsorption, achieving over 98% particulate filtration and ∼92% oil aerosol removal, while exhibiting antibacterial activity (Yu et al., 2023). β-PVDF/BaTiO_3_ ultrafiltration membranes prevent fouling, enhance pathogen and oil rejection, and extend lifespan via vibration and *in situ* ROS generation (Tan et al., 2022). Lead-free metal oxides, semiconductor composites, and organic piezoelectric polymers produce bactericidal ROS under flow or mechanical stimulation, enabling chemical-free disinfection (Meng et al., 2025). These membranes integrate sieving, electrostatics, antifouling, and ROS sterilization, representing next-generation, energy-efficient, adaptable filtration platforms.

## 4 Challenges and future perspectives

Despite the remarkable promise of piezoelectric sterilization as a sustainable and chemical-free antimicrobial strategy, several challenges must be addressed before it can achieve widespread adoption in clinical, industrial, and environmental settings. One of the foremost challenges lies in material optimization and standardization. A wide variety of piezoelectric materials have been explored, yet their antimicrobial efficiency, ROS generation capacity, and cytocompatibility vary considerably. Strategies such as doping, defect engineering, and nanostructuring enhance performance but may also introduce toxicity or compromise long-term stability. Standardized evaluation protocols are urgently needed to benchmark efficiency and ensure safety across diverse material systems (Gómez et al., 2018).

Biocompatibility and cytotoxicity represent another critical concern. While ROS generation is central to microbial inactivation, uncontrolled production risks collateral damage to mammalian cells and tissues. This is particularly problematic for applications in implants, wound dressings, or tissue scaffolds. Future efforts must focus on tailoring materials for selective antimicrobial activity while minimizing harm to host cells. For example, it has been demonstrated by Roy *et al.* that a biomimetic piezoelectric nanocomposite not only affects MRSA-induced bacterial infection through ROS production but also leads to the setup of an anti-inflammatory environment through macrophage repolarization for tissue repair (Roy et al., 2025). Moreover, a key safety issue for piezoelectric materials in chronic biomedical settings is ion leaching under long-term electromechanical stress. While doped bio-ceramics have shown good *in vivo* integration over ∼3 months with no major organ toxicity, there are virtually no studies for BFO or doped perovskites under repeated mechanical/electrical cycling. Data quantifying long-term release of Bi, Fe, or dopants, and tracking their accumulation in organs, are absent. Microstructural damage, pH shifts, inflammation, and high dopant levels could accelerate leaching. Future work must include long-term *in vitro* and *ex vivo* leaching under fatigue, plus *in vivo* biodistribution. In the meantime, any estimates suggest that worst-case ion release might remain below known toxicity thresholds, but this remains to be experimentally confirmed (Devi et al., 2018).

In terms of device integration and scalability, incorporating piezoelectric materials into practical platforms, such as coatings for surgical instruments, implantable devices, or water purification systems, presents challenges in adhesion, flexibility, and mechanical endurance. Additionally, large-scale synthesis of nanostructured or doped piezoelectric materials with consistent reproducibility remains a bottleneck to commercialization.

Energy efficiency and stimulation methods also require careful consideration. Current approaches rely on ultrasound, vibration, or fluid flow, which may increase energy costs or induce unwanted heating. Optimizing these stimulation modes, or coupling piezoelectricity with complementary modalities like photocatalysis or magnetism, could improve efficacy while reducing energy demand. Looking ahead, future perspectives highlight exciting opportunities. Development of biodegradable polymers, bio-inspired composites, and flexible membranes promises new generations of self-sterilizing coatings for implants, wearable devices, and wound dressings. The natural biomolecules (collagen, glycine, chitin) and synthetic biodegradable polymers (PLLA and PLA) combine mechanically induced electromechanical coupling with the ability to degrade safely in physiological environments, thereby reducing risks associated with chronic implants or removal surgery. While their piezoelectric coefficients currently lag those of ceramic counterparts, processing strategies such as dipole alignment, crystallinity control, and nanocomposites are improving their performance. Importantly, for applications such as self-powered hydrogel patches for wound healing, biodegradable piezoelectric materials offer the promise of delivering localized electrical stimulation and self-resorption post-therapy, thus enhancing safety, compatibility, and regulatory acceptability (Vannozzi et al., 2025). However, there are still some important hurdles to overcome. Making piezoelectric materials that are both soft and flexible, yet generate enough stimulation, is challenging. It is also essential to know exactly how and when they will break down in the body, and whether any by-products might cause harm. On top of that, we need dependable ways to measure how well they are working once implanted in real biological environments. In the future, wound-healing patches could greatly benefit from using biodegradable piezoelectric layers or fillers, but it will be key to design them so they deliver the right level of stimulation and degrade in a safe, predictable way.

Integration with hydrogels and electroactive scaffolds could create multifunctional systems that both support tissue regeneration and resist microbial colonization. Beyond healthcare, piezoelectric sterilization holds potential in environmental remediation, including antimicrobial water purification membranes and food packaging technologies. The convergence of piezoelectric platforms with artificial intelligence and smart sensing systems offers another frontier. The devices are capable of real-time pathogen detection and on-demand sterilization. For example, threshold-activated piezoelectric systems enable real-time microbial monitoring and autonomous sterilization on implants and catheters. Using interdigital electrode arrays, changes in impedance detect biofilm formation, triggering on-demand bioelectric effects that reduce *E. coli* coverage by ∼75% (Subramanian et al., 2017). Self-powered piezoelectric nanogenerators, such as PVDF hydrogels (Du et al., 2020) or ZnO films (Wan et al., 2021), convert mechanical or physiological motion into electricity to simultaneously sense microbial activity and activate localized ROS generation or electrical disruption once thresholds are exceeded. This feedback-controlled approach eliminates external power needs and manual intervention, enhancing antimicrobial efficiency, minimizing off-target effects, and offering a foundation for next-generation smart implants and wound dressings. By addressing challenges in material safety, scalability, and device design, piezoelectric sterilization can transition from a promising laboratory innovation into a practical, intelligent, and sustainable solution to global antimicrobial resistance.

## 5 Conclusion

Piezoelectric materials are emerging as a promising chemical-free and non-thermal sterilization strategy, generating localized electric charges and ROS under mechanical stimulation. This approach minimizes resistance development, toxicity, and material degradation, making it ideal for biomedical implants, surgical tools, wound dressings, and biofilm control. Antimicrobial action relies on both direct membrane disruption and ROS-mediated oxidative stress. Advances in defect engineering, nanostructuring, and doping have enhanced charge separation and controllable ROS production. Challenges remain in biocompatibility, long-term stability, and standardized evaluation, but smart coatings, biodegradable polymers, and multifunctional nanocomposites offer potential for scalable, intelligent, and sustainable piezoelectric sterilization solutions.
